# Development and evaluation of a mental health recovery priority measure for cross-cultural research: global INSPIRE

**DOI:** 10.1007/s00127-025-02946-9

**Published:** 2025-07-15

**Authors:** Yasuhiro Kotera, Akemi Hara, Christopher Newby, Yuki Miyamoto, Akihiko Ozaki, Yumna Ali, Pamela Clinton, Simon Felix, Catherine John, Natthapon Inta, Mike Slade

**Affiliations:** 1 School of Health Sciences, University of Nottingham, Nottingham, NG7 2TU UK; 2 Center for Infectious Disease Education and Research, The University of Osaka, Suita, 565-0871 Japan; 3 Department of Social Sciences, Azerbaijan University, Baku, AZ1007 Azerbaijan; 4 Medical Governance Research Institute, Tokyo, 108-0074 Japan; 5 School of Medicine, University of Nottingham, Nottingham, NG7 2UH UK; 6 Graduate School of Medicine, the University of Tokyo, Tokyo, 1130033 Japan; 7 Breast and Thyroid Surgery, Jyoban Hospital of Tokiwa Foundation, Iwaki, 972-8322 Japan; 8 Department of Psychology, Hazara University-Mansehra, Dhodial, 21120 Mansehra, Khyber Pakhtunkhwa Pakistan; 9 Department of Psychology, University of Bordeaux, Bordeaux, 33000 France; 10 Florence Nightingale Faculty of Nursing, Midwifery & Palliative Care, King’s College London, London, SE1 8WA UK; 11 Princess Agrarajakumari Faculty of Nursing, Chulabhorn Royal Academy, Bangkok, 10210 Thailand; 12 Faculty of Nursing and Health Sciences, Nord University, Postbox 474, Namsos, 7801 Norway

**Keywords:** Mental health recovery, Personal recovery, Cross-cultural validation, Scale development and validation, Recovery priorities

## Abstract

**Purpose:**

This study developed and evaluated a new personal recovery scale—Global INSPIRE—in English and Japanese, and compared responses between Japan and the UK. Personal recovery—living a satisfying and meaningful life despite mental health challenges—has gained attention in mental health. The widely-used CHIME framework identifies five recovery processes: Connectedness, Hope, Identity, Meaning, and Empowerment. Full INSPIRE evaluates whether service users feel supported in each CHIME process. However, emerging cross-cultural evidence indicates differences in the priority placed on each CHIME domain. No tool exists to assess recovery priorities, hindering cross-cultural research.

**Methods:**

The 20-item self-completed Global INSPIRE was adapted from the Full INSPIRE to assess recovery priorities of both service users and non-service users. Participants in the UK (*n* = 512) and Japan (*n* = 507) completed the Global INSPIRE at baseline and two-week follow-up. Psychometric properties were evaluated, including reliability, test-retest stability, and discriminant validity with two established recovery scales—CORE-10 and QPR-15. Cross-national differences in recovery priorities were also examined.

**Results:**

Both versions showed excellent reliability (α = 0.91 UK; 0.97 Japan), test-retest stability (4/5 processes UK, 5/5 Japan), and acceptable model fit (CFI = 0.80 UK; 0.85 Japan). Measurement invariance supported configural (CFI = 0.89) and metric invariance (CFI = 0.89) but not scalar invariance (CFI = 0.83). Discriminant validity was supported by weak correlations with CORE-10 and QPR-15. UK participants prioritised Hope, Meaning, and Empowerment, while Japanese participants prioritised Identity.

**Conclusion:**

Global INSPIRE is a reliable and culturally adaptable tool for assessing distinct recovery priorities. Future research should explore demographic differences and expand testing across diverse cultural contexts.

**Supplementary Information:**

The online version contains supplementary material available at 10.1007/s00127-025-02946-9.

## Introduction

Personal recovery in mental health refers to living a meaningful, satisfying, and fulfilling life, even in the presence of mental health challenges [1]. While clinical recovery focuses on symptom reduction and functional improvement, personal recovery emphasises subjective experiences, including hope, identity, and connectedness [2]. Over the past two decades, personal recovery, hereafter ‘recovery’, has gained increasing attention in mental health research and practice, and is now embedded in national mental health policies across many countries. For example, in the United Kingdom (UK), the National Health Service emphasises recovery-oriented care, promoting collaborative care planning and peer support programs [3]. In Canada, the Mental Health Commission of Canada has developed the Guidelines for Recovery-Oriented Practice, which serve as a comprehensive reference for integrating recovery principles into mental health services nationwide [4]. Australia has incorporated recovery principles into the National Mental Health Strategy [5]. The Substance Abuse and Mental Health Services Administration in the United States of America identifies recovery as a guiding principle, highlighting self-direction and person-centred care [6].

This orientation is also found in non-Anglophone and non-Western countries. For example, recovery-oriented mental health care was introduced in Japan in 1998 and has since become a key focus of services [7]. In 2004, Japan’s Ministry of Health, Labour and Welfare proposed shifting from hospital-based treatment to community-based care, leading to services such as day care, psychiatric home-visit nursing, and employment transition support [8]. Around the same time, Tojisya Kenkyu [当事者研究], a self-empowerment programme, emerged [9]. Recovery-focused approaches from the West, including Assertive Community Treatment [10], Wellness Recovery Action Plan [11], and Recovery Colleges [12], are now widely practiced. Psychometric tools to assess recovery outcomes and attitudes have also been validated in Japanese contexts [13].

The evidence base for recovery is growing. Meta-syntheses identified that personal recovery can transform despair to well-being [14], and is relevant to diverse population groups and mental health problems [15]. A qualitative evidence synthesis reported that personal recovery helps overcome stigma and establish meaning in life [16].

A systematic review identified the five processes of personal recovery, and developed the CHIME framework: Connectedness, Hope, Identity, Meaning, and Empowerment [17]. The CHIME framework is the most established framework of personal recovery [18], and used in international guidance [19]. However, knowledge gaps exist in development of standardised measures, and cross-cultural understanding [20].

The Full INSPIRE is a service user-rated 27-item standardised measure (where “INSPIRE” is a name rather than an acronym), developed to assess how well mental health services support recovery [21]. It is based on the CHIME framework, and comprises two subscales: the Support subscale (20 items) assesses the extent to which a service user experiences recovery support from their mental health worker, and the Relationship subscale (7 items) assesses the quality of the therapeutic relationship between the service user and the worker. To reduce respondent burden, the Brief INSPIRE was developed as a shorter version, focusing only on the Support subscale, and comprising five items that capture key aspects of recovery support [22]. A further adaptation, the Brief INSPIRE-O (“O” for outcome), shifts the focus from support received to the service user’s own recovery progress, providing a brief five-item assessment of personal recovery outcomes rather than support processes [22]. Thus, while Full INSPIRE provides a comprehensive assessment of both support and relationship quality, Brief INSPIRE focuses narrowly on perceived support, and Brief INSPIRE-O measures recovery outcomes from the service user’s perspective. INSPIRE has been translated into 27 languages (researchintorecovery.com/inspire), with validation studies completed in Danish [22], Italian [23], Japanese [7] and Swedish [24]. However, no tool currently exists to assess recovery priorities — that is, to identify which CHIME processes matter most to respondents. Without understanding recovery priorities, interventions can be less effective or even harmful; for example, individuals who want to focus on Identity through interventions such as self-reflection may find it distracting or unhelpful to receive Connectedness-focused interventions such as group activities.

There is preliminary evidence that the priority attached to different recovery processes differs across cultures. Despite the strong endorsements of personal recovery across cultures [25], its understanding is diverse. Autonomy is often prioritised in Anglo-Saxon individualistic countries, whereas community and social relationships are foregrounded in more collectivistic countries [26]. In Japan, for instance, compassion for others is considered fundamental to personal recovery [27]. Similarly, in Arab cultures, traditional values and religious beliefs are deeply influential in shaping recovery experiences [28]. In Brazil, recovery tends to focus on community involvement and fulfilling social roles [29]. In Poland, the importance of close family relationships alongside a strong sense of national identity suggests that recovery approaches may need to emphasise both personal growth and community belonging [30]. In Spain [31] and South Africa [32], relational aspects of recovery are especially prominent, while in Taiwan, empowerment and social support emerge as key recovery priorities [33]. These studies highlight that recovery priorities differ across cultures, yet no tool currently exists to assess recovery priorities.

This study focuses on the UK and Japan for several reasons. First, both countries have actively embraced recovery-oriented approaches at a national policy level, with well-established services and practices promoting personal recovery [3]. Second, the UK and Japan have comparable economic development levels and high standards of healthcare quality, providing a relatively stable basis for cross-cultural comparison without confounding effects from major systemic disparities [34]. Third, the two countries represent culturally distinct contexts: the UK is characterised by individualism, indulgence, acceptance of uncertainty, and a short-term orientation, whereas Japan is characterised by collectivism, restraint, uncertainty avoidance, and a long-term orientation [35]. These contrasting cultural profiles provide a valuable opportunity to explore cross-cultural differences may shape recovery priorities.

The aim of this study was to develop, evaluate and apply a new mental health recovery priority measure, called Global INSPIRE. The three objectives were to develop and evaluate the psychometric properties of Global INSPIRE in (1) English and (2) Japanese, and then (3) to conduct a proof-of-concept study by comparing scores to explore cross-cultural differences in recovery priorities between the UK and Japan.

## Methods

### Development of global INSPIRE

Global INSPIRE was initially developed in English by adapting items from the Support subscale of the English Full INSPIRE, which comprises 20 items (four items per CHIME subscale) completed by service users to assess recovery support from a mental health worker. Each Full INSPIRE item includes two parts: (1) whether the item is important for the respondent’s recovery (rated Yes or No) and (2) if rated Yes, the extent of recovery support received from the worker on that item.

Global INSPIRE draws directly from the first part of each Full INSPIRE item with adaptation to include non-service users, who have never used mental health services. All items begin with “*An important part of my recovery is…*”, with example items such as *“…feeling supported by other people*” (Connectedness), *“…feeling hopeful about my future*” (Hope). To allow participation from non-service users, three items were modified: Item 3 “Having support from other people who use services” to “Having support from other people who live with mental health difficulties,” Item 6 “Believing that I can recover” to “Believing that I can recover (live a satisfying and hopeful life)”, and Item 9 “Feeling I can deal with stigma” to “Feeling I can deal with mental health stigma.” These adaptations ensured the scale’s relevance for both service users and non-service users while maintaining psychometric integrity.

The Full INSPIRE Support subscale demonstrated strong psychometric properties in a mental health service user sample (*n* = 92). It showed good convergent validity, with a correlation of 0.60 with the Satisfaction Index-Mental Health (SI-MH) [36]. Exploratory factor analysis confirmed a five-factor structure accounting for 85.1% of the variance, with Kaiser-Meyer-Olkin values ranging from 0.65 to 0.78 across the CHIME domains. The subscale also exhibited high internal consistency (α 0.82–0.85). Additionally, it demonstrated sensitivity to change, with only 8% of responses changing inconsistently when compared to the SI-MH.

The Global INSPIRE is designed for the general population. Items are rated on a five-point Likert scale (0 = “Not at all” to 4 = “Very much”). Subscale scores are calculated by summing four items, dividing by 16, and multiplying by 100, yielding scores from 0 (low priority) to 100. The Global INSPIRE and its manual are available from https://www.researchintorecovery.com/measures/inspire/.

The Japanese Global INSPIRE was developed by adjusting the wording of the Support subscale of the Japanese version of the Full INSPIRE [7] with YK and YM ensuring conceptual equivalence—meaning the measured concepts are consistent between English and Japanese [37]. The adjusted scale was piloted by nine Japanese-native speakers (five females and four males; four service users) to ensure it was clear and easy to understand in Japanese. Their feedback was incorporated into the measure (e.g., the definition of personal recovery—“living a satisfying and hopeful life”—was added every time “recovery” was mentioned rather than only at the first mention) to finalise the Japanese version of the Global INSPIRE.

## Evaluation of global INSPIRE

An a priori power analysis was conducted to determine the required sample size for the study. For test-retest reliability analysis, a paired t-test was used, assuming a small effect size (Cohen’s d = 0.2), α = 0.05 (5% error rate), and 80% power (G*Power version 3.1.9.7). The null hypothesis assumes no difference between time points, while the alternative hypothesis assumes a statistically significant difference between time points in test-retest reliability scores. The analysis indicated that a minimum of 198 participants per country was required to detect a statistically significant effect.

To ensure sufficient power for confirmatory factor analysis (CFA), which requires larger samples to test model fit and latent factor structures [38], we recruited 500 participants per country. Recent methodological guidance recommends basing CFA sample size on the complexity of the model, particularly the number of estimated parameters and degrees of freedom, rather than on a simple ratio of participants to items [39]. Sample sizes of at least 300–500 are generally considered sufficient to achieve stable fit indices and parameter estimates for models of moderate complexity [40]. Our sample size of 500 participants per country therefore provided adequate power to support the planned analyses. All participants completed assessments at baseline and two-week follow-up, enabling robust evaluation of test-retest reliability and construct validity.

UK participants were recruited via Prolific and Japanese participants via Cross Marketing, both online platforms. Eligible participants (aged 18+, residing in the UK or Japan, and fluent in English or Japanese) received study information and consent forms. Upon consent, they completed demographic questions and the Global INSPIRE. The same participants completed the Global INSPIRE again after two weeks. Japanese participants were recruited after the UK ones, ensuring that the gender balance and age splits were identical to the UK participants.

Internal consistency and reliability were assessed by ordinal alphas, Cronbach’s alphas (≥ 0.90 as excellent; 0.80–0.89 as good; 0.70–0.79 as acceptable), and item-total correlations (≥ 0.40 as very good; 0.30–0.39 as adequate). Test-retest reliability was assessed using paired t-tests. The fit of the five CHIME sub-scales was assessed using confirmatory factor analysis. Four fit indices were calculated: Comparative Fit Index (CFI), Tucker-Lewis Index (TLI) (≥ 0.95 as excellent; 0.90–0.94 as acceptable for both CFI and TLI), Root Mean Square Error of Approximation (RMSEA) (≤ 0.05 as excellent; 0.06–0.08 as acceptable; 0.08–0.10 as marginal with 90% Confidence Interval (CI) falling below 0.08), and Standardised Root Mean Square Residual (SRMR) (≤ 0.08 as acceptable; ≤ 0.05 as excellent) [41]. Considering the multi-cultural composite of the UK sample (26.4% in ethnic minorities; see Table 1), looser acceptable thresholds were applied: 0.80 for CFI and TLI, 0.10 for RMSEA, and 0.12 for SRMR [42]. Cultural and linguistic diversity can introduce additional variance that reflects genuine subgroup differences rather than model misspecification [43]. In such contexts, conventional strict thresholds (e.g., CFI ≥ 0.95) may underestimate the adequacy of theoretically sound models [44]. Therefore, applying slightly relaxed criteria is a recognised approach in cross-cultural validation studies to ensure fair assessment of model fit in heterogeneous samples [45]. To further improve model fit, a revised CFA model was also tested with the addition of several theoretically justifiable correlated residuals within the Identity and Empowerment subscales. Full details are provided in Supplementary Material 1.

In addition to traditional CFA, a bifactor CFA model without orthogonal constraints was estimated to assess the extent to which a general recovery factor explains shared variance across all 20 items, relative to the five specific CHIME domains. In this structure, each item was allowed to load onto both a general factor and its respective CHIME dimension, while correlations among latent variables were freely estimated. To evaluate the dimensionality captured by the bifactor model, Explained Common Variance (ECV) was computed. The ECV reflects the proportion of common variance attributable to the general factor, providing insight into whether the scale functions predominantly as unidimensional or multidimensional. ECV values closer to 1.00 indicate greater unidimensionality; values above 0.70 are typically interpreted as supporting a primarily general-factor structure. Additionally, measurement model reliability to examine latent variables (α ≥ 0.70 as acceptable), and convergent validity to assess indicator agreement (Average Variance Extracted (AVE) ≥ 0.50 as acceptable) were examined [46].

The measurement invariance of the Global INSPIRE scale was evaluated using Multi-Group Confirmatory Factor Analysis (MGCFA) with aggregated data from the UK and Japan. Given the ordinal nature of the data, the Weighted Least Squares Mean and Variance adjusted (WLSMV) estimator was applied to account for the ordered categorical responses. A hierarchical approach was used to assess invariance across three levels. First, configural invariance tested whether the factor structure (e.g., number of factors and item loadings) was consistent across groups. Second, metric invariance evaluated whether factor loadings were equal, ensuring the constructs were measured equivalently. Finally, scalar invariance assessed whether item intercepts were comparable, enabling latent mean comparisons between the two countries. Model fit was assessed using Chi-Square, CFI and TLI (≥ 0.90 as acceptable), RMSEA (≤ 0.08 as acceptable), and SRMR (≤ 0.08 as acceptable) indices. This approach aimed to validate the scale’s comparability across cultural and temporal contexts, providing evidence of its cross-cultural applicability.

Discriminant validity was evaluated using the same UK sample recruited via Prolific two months after the baseline assessment to minimise memory effects and reduce participant fatigue. Participants completed the Global INSPIRE alongside the Clinical Outcomes in Routine Evaluation-10 (CORE-10) [47] and the Questionnaire about the Process of Recovery-15 (QPR-15) [48]. Discriminant validity was assessed through three methods: (1) Spearman correlation analysis, where correlations below 0.40 indicate distinct constructs [39]; (2) CFA comparing single-factor and three-factor models, with a better fit for the three-factor model supporting distinctness; and (3) comparison of AVE with squared correlations (r²) using the Fornell-Larcker criterion [49], where AVE values greater than r² indicate distinct constructs.

## Comparison of UK and Japan data

To explore the use of the Global INSPIRE for comparing recovery priorities between countries, Global INSPIRE scores were compared between the UK and Japan using Analysis of Variance (ANOVA). If the results were significant, Tukey Honest Significant Difference (HSD) Tests were used to identify which CHIME components were significantly different between the two samples. All statistical analyses were conducted using R Studio (version 4.4.1). Statistical significance was established at *p* < 0.05.

## Results

The 20-item English Global INSPIRE was completed at baseline by 512 participants, of whom 485 (94.7%) completed the two-week follow-up. The 20-item Japanese Global INSPIRE was completed by 507 participants at baseline and two-week follow-up. Participant characteristics are shown in Table 1.

**Table 1:** Participant characteristics

	UK (*n* = 512) *n* (%)	Japan (*n* = 507) *n* (%)
**Age (years)**	41 ± 13.31	42 ± 13.91
**Gender** Female	322 (62.9)	319 (62.9)
Male	187 (36.5)	187 (36.9)
Non-binary/prefer not to say	≤ 5	≤ 5
**Ethnicity** White British Other White Asian/Asian British Black/African/Caribbean/Black British Mixed/Multiple ethnic groups Arab Other/Prefer not to say	377 (73.6%) 38 (7.5%) 45 (8.8%) 28 (5.5%) 18 (3.5%) ≤ 5 ≤ 5	
**Sexual** Straight/Heterosexual	445 (86.9%)	435 (85.8%)
Gay or Lesbian	16 (3.1%)	≤ 5
Bisexual	43 (8.4%)	16 (3.2%)
Other/Prefer not to say/Unknown	8 (1.6%)	18 (3.6%)
**Mental health service experience**		
Yes	257 (50.2%)	109 (21.5%)
*Less than 1 year*	*118 (23.0%)*	*39 (7.7%)*
*1 to 5 years*	*83 (16.2%)*	*31 (6.1%)*
*More than 5 years*	*56 (10.9%)*	*39 (7.7%)*
No	255 (49.8%)	398 (78.5%)

Note: For categories with counts of 5 or fewer, values are reported as ≤ 5 to prevent identifiability. Ethnicity was not assessed for the Japanese sample, as the population is predominantly of Asian ethnicity

Evaluation of the psychometric properties of the English and Japanese Global INSPIRE are shown in Table 2.

**Table 2:** Results of global INSPIRE validation in the UK and Japan

		UK				Japan			
(a) Internal Consistency and Reliability	**Measure**	**Value**		**95% CI**		**Value**		**95% CI**	
	Ordinal α	0.94		[0.89, 0.97]		0.97		[0.95, 0.99]	
	Cronbach’s α	0.91		[0.90, 0.91]		0.96		[0.96, 0.97]	
	Item-Total Correlations	0.47–0.70^†^		_		0.66–0.83^†^		_	
(b) Test-Retest Reliability	**Process**	**t-value**	**p-value**	**MD**	**95% CI**	**t-value**	**p-value**	**MD**	**95% CI**
	Connectedness	−1.27	0.2	−1.79	[−4.55, 0,97]	−0.35	0.73	−0.32	[−2.12, 1.48]
	Hope	−0.82	0.41	−0.89	[−3.03, 1.24]	−0.99	0.32	−0.92	[−2.76, 0.91]
	Identity	1.04	0.3	1.5	[−1.34, 4.35]	−0.43	0.67	−0.39	[−2.18, 1.40]
	Meaning	−1.56	0.12	−1.63	[−3.69, 0.42]	0.34	0.73	0.31	[−1.47, 2.09]
	Empowerment	−2.28	0.02*	−2.52	[−4.68, 0.35]	−0.09	0.93	−0.07	[−1.75, 1.60]
(c) CFA	Chi-square (p-value)	< 0.001				< 0.001			
Fit Indices	CFI	0.80				0.95			
	TLI	0.77				0.93			
	RMSEA [90%CI]	0.11 [0.10, 0.11]				0.07 [0.07, 0.08]			
	SRMR	0.12				0.04			
(d) Bifactor CFA Fit Indices	Chi-square (p-value)	< 0.001				< 0.001			
	CFI	0.99				1.00			
	TLI	0.99				1.00			
	RMSEA [90%CI]	0.05 [0.05, 0.06]				0.04 [0.03,0.04]			
	SRMR	0.04				0.02			
	ECV	0.31				0.71			
Measurement Model Reliability	**Latent Variable**	**Cronbach’s Alpha**							
	Connectedness	0.74				0.85			
	Hope	0.85				0.89			
	Identity	0.70				0.81			
	Meaning	0.77				0.87			
	Empowerment	0.76				0.84			
Convergent Validity	**Latent Variable**	**Average Variance Extracted**							
	Connectedness	0.41				0.58			
	Hope	0.59				0.66			
	Identity	0.51				0.51			
	Meaning	0.47				0.62			
	Empowerment	0.43				0.57			
Multi-Group Model Fit Indices for Measurement Invariance (UK-Japan aggregated)	**Fit Indices**	**Configural Model**		**Metric Model**		**Scalar Model**			
	χ²	3025.73		25074.97		25074.97			
	p-value	< 0.001		< 0.001		< 0.001			
	CFI	0.89		0.89		0.83			
	TLI	0.87		0.87		0.81			
	RMSEA [90%CI]	0.09 [0.09, 0.10]		0.09 [0.09, 0.09]		0.11 [0.11, 0.11]			
	SRMR	0.08		0.06		0.09			
CFA = Confirmatory Factor Analysis; CI = Confidence Interval; MD = Mean Difference; CFI = Comparative Fit Index; TLI = Tucker-Lewis Index; RMSEA = Root Mean Square Error of Approximation; SRMR = Standardised Root Mean Square Residual. ^†^ = Range of correlations across all 20 items.									

## Internal consistency

The English Global INSPIRE had excellent internal consistency. Both ordinal alpha (α = 0.94, 95% CI [0.89, 0.97]) and Cronbach’s alpha (α = 0.91, 95% CI [0.90, 0.91]) confirmed strong reliability. The narrow confidence intervals (CIs) indicate that deleting any single item had minimal impact on the overall reliability. Item-total correlations ranged from 0.47 to 0.70, with item 3 (Having support from other people who live with mental health difficulties) showing the lowest correlation (0.47) and item 15 (Rebuilding my life after difficult experiences) the highest (0.70).

The Japanese Global INSPIRE had exceptional internal consistency, with both ordinal alpha (α = 0.97, 95% CI [0.95, 0.99]) and Cronbach’s alpha (α = 0.96, 95% CI [0.96, 0.97]) indicating excellent reliability. While the high Cronbach’s α could suggest redundancy, the item-total correlations (0.66–0.83) confirm that all items contribute meaningfully to the scale, with Q11 showing the lowest correlation (0.66) and Q6 the highest (0.83). Deleting any single item had minimal impact on overall reliability, suggesting each item plays a vital role in the scale’s reliability. These findings demonstrate robust internal consistency of the Japanese version.

### Test-retest reliability

Four of five CHIME sub-scales in the English Global INSPIRE demonstrated test-retest reliability. Paired t-tests comparing the baseline and two-week follow-up revealed that no significant differences were found between the two time points for Connectedness, Hope, Identity, and Meaning (*p* > 0.05). A significant decrease was observed for Empowerment (t(481)=−2.28, mean difference − 2.52, 95% CI −4.68 to −0.35, *p* < 0.05), indicating that participants reported lower Empowerment scores at follow-up.

All five CHIME sub-scales in the Japanese Global INSPIRE demonstrated test-retest reliability. Paired t-tests revealed no statistically significant differences for any of the CHIME dimensions. The mean differences between time points were close to zero, ranging from − 0.92 to 0.31, and the 95% CIs for all dimensions included 0, indicating minimal measurement error and temporal stability.

## Sub-scale structure

An acceptable model fit was indicated for the English Global INSPIRE (See the path diagram in Supplementary Material 2). The CFI (0.80) and TLI (0.77) approached the acceptable threshold of 0.80 and SRMS (0.12) met the threshold, while the RMSEA (0.11) slightly exceeded the 0.10 threshold. An adjusted CFA model improved the fit indices, with CFI increasing to 0.86, TLI to 0.83, and RMSEA decreasing to 0.09. However, these improvements remained marginal, and overall model fit did not reach conventional cutoffs for excellent fit. Measurement model reliability demonstrated acceptable internal consistency for most latent variables, with Cronbach’s Alpha values ranging from 0.70 to 0.85. However, AVE values revealed potential convergent validity concerns for Connectedness (AVE = 0.41), Meaning (AVE = 0.47), and Empowerment (AVE = 0.43), which fell below the 0.50 threshold. These findings suggest the model reasonably captures the underlying data structure, though further refinements may improve validity.

An acceptable model fit was indicated for the Japanese Global INSPIRE (See the path diagram in Supplementary Material 2). The CFI (0.95) and TLI (0.93) exceeded the acceptable threshold of 0.90, while the RMSEA (0.07) with a 90% CI [0.07, 0.08] fell within the acceptable limit (≤ 0.08), with its confidence interval also below 0.08. The SRMR (0.04) met the excellent fit threshold (≤ 0.05). Measurement model reliability demonstrated strong internal consistency, with Cronbach’s Alpha values ranging from 0.81 to 0.89 across all latent variables. Additionally, AVE values were satisfactory across constructs, ranging from 0.51 to 0.66, thereby meeting the recommended threshold (≥ 0.50).

The bifactor model in the UK sample demonstrated substantially improved model fit compared to the original five-factor CFA (Bifactor: CFI = 0.99, TLI = 0.99, RMSEA = 0.05), indicating that a strong general factor underlies item responses. In the Japan sample, while the original CFA already showed acceptable fit, the bifactor model still yielded superior fit (CFI = 0.999, TLI = 0.999, RMSEA = 0.04), suggesting that the general factor is also meaningful in this context. The ECV—which reflects how much variance is accounted for by the general recovery factor as opposed to the five CHIME domains—was 0.31 in the UK sample (i.e., the general factor explained 31% of the common variance) and 0.71 in the Japanese sample. This pattern indicates that, while both samples support the presence of a general recovery priority dimension, the UK data retain substantial multidimensionality across the CHIME subscales, whereas the Japanese data are more strongly driven by the overarching recovery construct.

## Measurement invariance

The measurement invariance analysis supported configural invariance (CFI = 0.89, TLI = 0.87, RMSEA = 0.09, SRMR = 0.08, *p* < 0.001), indicating that the factor structure was consistent across the UK and Japanese groups and time points. Metric invariance (CFI = 0.89, TLI = 0.87, RMSEA = 0.09, SRMR = 0.06, *p* < 0.001) was supported, indicating that the factor loadings were equivalent across groups. However, scalar invariance (CFI = 0.83, TLI = 0.81, RMSEA = 0.11, SRMR = 0.09, *p* < 0.001) was not fully supported, indicating differences in item intercepts, likely reflecting cultural variations in how recovery priorities are interpreted. These findings suggest that comparisons of factor relationships between groups are valid, but direct comparisons of mean scores should be interpreted cautiously.

### Discriminant validity

Correlation analysis revealed all CHIME dimensions were weakly associated with both the total CORE-10 scores (*r* = −0.21 to −0.29) and the total QPR-15 scores (*r* = 0.35 to 0.39): all *r* < 0.40, indicating the distinctness of the Global INSPIRE. Likewise, the CFA showed that the three-factor model significantly outperformed the single-factor model (Δχ² = 1463, *p* < 0.001), supporting the multidimensional structure. The three-factor model demonstrated better fit indices (χ² = 1054.82, CFI = 0.92, TLI = 0.91, RMSEA = 0.06, SRMR = 0.06) compared to the single-factor model (χ² = 2517.84, CFI = 0.74, TLI = 0.72, RMSEA = 0.11, SRMR = 0.10). Lower AIC (Single-factor 40853.40 > Three-factor 39396.38) and BIC values (Single-factor 41095.10 > Three-factor 39650.16) also favored the three-factor model. These findings confirm that distinguishing between QPR-15, CORE-10, and CHIME scales provides a more accurate model fit.

The discriminant validity analysis showed mixed results. A comparison between CORE-10 and QPR-15 lacked discriminant validity (*r*² = 0.62 > AVE_CORE10 = 0.48; AVE_QPR15 = 0.56), indicating substantial overlap. However, comparisons between the Global INSPIRE and CORE-10 (*r*² = 0.18 < AVE_Global INSPIRE = 0.60; AVE_QPR15 = 0.56), and the Global INSPIRE and QPR-15 (*r*² = 0.05 < AVE_CORE10 = 0.48; AVE_Global INSPIRE = 0.60) demonstrated strong discriminant validity, confirming conceptual distinction.

### UK-Japan comparison

Table 3 presents the results of ANOVA and Tukey HSD of the GLOBAL INSPIRE scores between the two samples.

**Table 3:** ANOVA and Tukey HSD between the UK and Japan

	Dimension	F-value		*p*-value
ANOVA	Connectedness	2.05		0.15
	Hope	435.00		< 0.001
	Identity	69.56		< 0.001
	Meaning	230.40		< 0.001
	Empowerment	142.50		< 0.001
	Dimension	MD	95% CI	p-value
Tukey HSD for post-hoc comparisons^	Hope	17.80	[16.12, 19.47]	< 0.001
	Identity	−7.97	[−9.84, −6.09]	< 0.001
	Meaning	12.86	[11.20, 14.52]	< 0.001
	Empowerment	9.93	[8.29, 11.56]	< 0.001
^Comparing the UK data to the Japanese data (i.e., positive values indicate UK was higher). MD = Mean Difference; CI = Confidence Interval.				

ANOVA identified significant differences in all but Connection (*p* = 0.15). The Tukey HSD results revealed that scores for Hope were higher in the UK compared to Japan, with a mean difference of 17.80. In contrast, Identity scores were lower in the UK, showing a mean difference of −7.97. Similarly, Meaning and Empowerment were both higher in the UK, with mean differences of 12.86 and 9.93, respectively.

## Discussion

This study developed and evaluated the Global INSPIRE in UK and Japanese populations, focusing on validation, cross-cultural applicability, and comparative analyses. Both versions demonstrated high internal consistency, reliability, and acceptable model fit. Cross-cultural testing supported metric invariance, enabling comparisons of factor relationships across groups, although scalar invariance was not achieved. Discriminant validity of the Global INSPIRE is high, indicating the scale is measuring unique constructs that the existing established recovery measures do not capture. Significant differences in the two datasets were observed, with higher Identity scores in Japan and higher Hope, Meaning, and Empowerment scores in the UK.

The findings from this study provide important insights into the psychometric properties of the Global INSPIRE scale and its cross-cultural and domestic applicability. Both the UK and Japanese versions demonstrated strong internal consistency and test-retest reliability, confirming the scale’s stability over time. This is a significant contribution to the expanding global mental health recovery landscape. Domestically, the Global INSPIRE offers a valuable tool for identifying what is most important to service users, enabling services to tailor their interventions to meet these specific needs. For instance, if connectedness is highlighted as a priority, services could focus on connectedness-oriented interventions such as peer support programmes or social skills training. Alternatively, if identity emerges as a key focus, services could provide support in building positive identity through targeted programmes. On a global scale, the scale’s validation supports cultural adaptation of recovery-oriented mental healthcare, which has increasingly become important [50]. For example, Recovery Colleges (RCs), a recent mental health innovation, are learning-based mental health recovery support systems [51]. Since the first RC opened in England in 2009, 221 RCs have opened in 28 countries across cultures [52]. Validating the Global INSPIRE in other languages and using the validated scales in different cultural regions will enable wider cross-cultural comparisons, helping to identify key recovery processes across different cultures. Currently, validations in other languages (e.g., French, Urdu, Thai) and contexts (e.g., Ireland) are underway. The Global INSPIRE can be a helpful tool to support the global and domestic expansion of mental health recovery.

The finding from the bifactor CFAs was that the UK data exhibited greater multidimensionality across CHIME subscales, while the Japanese data were more strongly influenced by a single general factor. This pattern may suggest that UK participants were more attuned to identifying and differentiating their personal recovery priorities, giving greater emphasis to some CHIME domains over others—such as prioritising Hope or Empowerment over Connectedness or Meaning. In contrast, Japanese participants appeared to view the CHIME domains as more equally important, resulting in a flatter response profile across subscales. One possible contributing factor is cultural variation in response style: people in the UK may be more likely to use the full range of rating scales, including more extreme values, while people in Japan may prefer more moderate, balanced responses [53]. Such response patterns are well-documented in cross-cultural research and may partly explain the stronger unidimensionality observed in the Japanese data. These findings highlight the importance of considering both substantive interpretation and methodological context when examining cross-cultural differences in recovery priorities [54].

The decline in Empowerment in the UK sample warrants attention. We conducted a post-hoc analysis to examine whether this change differed between service users and non-service users. Paired t-tests revealed that among service users (*n* = 237), Empowerment scores significantly declined from baseline to follow-up (t(236) = −2.65, *p* = 0.009, mean difference = −4.14, 95% CI [−7.21, −1.07]), whereas no significant difference was found for non-service users (*n* = 245; t(244) = −0.59, *p* = 0.554, mean difference = −0.94, 95% CI [−4.08, 2.19]). This suggests that the observed change in Empowerment is not a general trend across all participants but is specific to those with lived experience of mental health services. Several factors may help explain this pattern. First, service users may have engaged in deeper self-reflection after the baseline assessment, leading to a response shift effect in which their understanding of empowerment evolved, resulting in more critical self-appraisals at follow-up [55]. Second, individuals engaged in services may hold higher expectations for recovery, and any perceived stagnation or lack of progress could lead to lower subsequent ratings of empowerment [56]. Third, cultural expectations in the UK that emphasise personal agency and achievement may intensify these effects for service users who feel pressure to demonstrate improvement [57].

The importance of addressing cultural variations was indicated by the model fit indices for the English version and unsupported scalar invariance. These issues may reflect the diverse demographics in the UK, where the proportion of ethnic minority groups has been increasing and is expected to grow further [58]. The wording in the Global INSPIRE, particularly for Connectedness, Meaning, and Empowerment, could be refined to better capture perspectives on personal recovery from diverse groups. Similarly, the measurement invariance analysis supported configural and metric invariance, suggesting that the factor structure and factor loadings were consistent across groups. However, scalar invariance was not fully supported, indicating differences in item intercepts for reasons such as UK-Japan cross-cultural differences in personal recovery. As a result, while comparisons of factor relationships (e.g., correlations and regressions) are valid, direct comparisons of mean scores should be interpreted cautiously. Nevertheless, we conducted exploratory comparisons of mean scores to provide preliminary insights into potential patterns and cultural differences in recovery priorities, recognising the need for further validation in future studies.

The UK-Japan comparisons highlighted notable cultural differences in recovery priorities. Higher Identity scores in Japan may reflect the current shift in Japanese society from traditionally collectivistic values to a more individualistic orientation. In collectivistic cultures such as Japan, the self-concept has historically been shaped by how individuals are perceived by others [59]. However, individualism has become more accepted in recent years, and having a positive identity is increasingly viewed as important [60]. In contrast, higher Hope, Meaning, and Empowerment scores in the UK may indicate a greater emphasis on optimism, purpose, and personal agency, which align with individualistic values that encourage proactive self-improvement and autonomy [61]. Beyond individualism, societal narratives around personal growth and self-actualisation may further explain these findings. In the UK, ideas rooted in humanistic psychology—such as Maslow’s emphasis on meaning-making and self-fulfilment—are prominent in education, therapy, and public discourse [62]. Moreover, philosophical and religious traditions, such as Protestant ethics and existentialist thought, emphasise individual responsibility and the pursuit of purpose, possibly encouraging greater prioritisation of Meaning-related recovery processes [63]. Thus, differences in recovery priorities may not solely reflect individualism versus collectivism but also broader societal frameworks that shape how individuals conceptualise hope, meaning, and empowerment in their lives.

Strengths of this study include its cultural adaptability, as it validates the Global INSPIRE in both English and Japanese, addressing cross-cultural recovery priorities and expanding its applicability to diverse contexts. The study employs rigorous sample sizes [38] and robust validation methods, including CFA, test-retest reliability, measurement invariance testing, and discriminant validity analysis, ensuring the scale’s reliability and conceptual distinctiveness. The discriminant validity findings confirmed that the Global INSPIRE measures unique constructs not captured by existing recovery measures, further establishing its utility. Additionally, the inclusion of two time points allowed for an assessment of temporal stability, further strengthening the evidence of reliability.

Four limitations are noteworthy. First, potential dependencies in the data structure may have arisen due to repeated measurements from the same participants. Although our longitudinal approach accounts for temporal dependencies between baseline and follow-up measurements, it does not fully address the nested nature of individual responses. Future research could incorporate multilevel modelling approaches to simultaneously account for both temporal and individual-level dependencies in cross-cultural longitudinal data. Second, the use of self-report measures may have introduced response biases, such as social desirability or recall bias, potentially affecting the accuracy of the data [64]. Future studies could consider complementing self-report data with qualitative methods or informant perspectives to enhance understanding. Third, recruitment strategies may have introduced sampling biases. Offering financial incentives might have attracted participants motivated primarily by monetary gain. Moreover, online recruitment methods may have excluded individuals who do not regularly use the internet or feel hesitant to engage in online studies. These factors may limit the generalisability of the findings and should be addressed in future research. Lastly, this study did not examine differences in recovery priorities across demographic variables, such as gender and ethnicity [65]. Further research is needed to explore these variations and assess their implications for recovery interventions.

## Conclusion

The Global INSPIRE demonstrated strong psychometric properties in both UK and Japanese samples, with high reliability, acceptable model fit, and robust discriminant validity, confirming it measures unique constructs not captured by existing recovery measures. While metric invariance supported valid comparisons of factor relationships across groups, the lack of scalar invariance highlights the need for caution when comparing mean scores. Notable cultural differences in recovery priorities emphasise the importance of cultural adaptability in mental health assessments. Future research should focus on refining the model, addressing convergent validity issues, and exploring demographic influences to enhance the scale’s applicability across diverse populations. The Global INSPIRE’s demonstrated adaptability and discriminant validity underscore its potential as a tool for guiding culturally responsive interventions and policy development in mental health recovery worldwide.

## Electronic supplementary material

Below is the link to the electronic supplementary material.

Supplementary Material 1

## Data Availability

The data are not publicly available due to ethical restrictions but can be shared upon reasonable request to the corresponding author.
