# Supplementary material for: Development and evaluation of a mental health recovery priority measure for cross-cultural research: global INSPIRE
Source: Soc Psychiatry Psychiatr Epidemiol. 2025 Jul 15;60(11):2695–706. doi: 10.1007/s00127-025-02946-9 (PMC12572008; doi:10.1007/s00127-025-02946-9)
Supplement: Supplementary file 1 — Supplementary Material 1 [file 127_2025_2946_MOESM1_ESM.docx]

**Supplementary Material 1: Fit Indices for Adjusted CFA**

| Fit Index | Value |
| --- | --- |
| CFI | 0.86 |
| TLI | 0.83 |
| RMSEA | 0.11 |
| SRMR | 0.08 |

CFI = Comparative Fit Index; TLI = Tucker-Lewis Index; RMSEA = Root Mean Square Error of Approximation; SRMR = Standardised Root Mean Square Residual

**Supplementary Material 2: Confirmatory factor analysis path diagram for UK data (top) and Japanese data (bottom)**

| **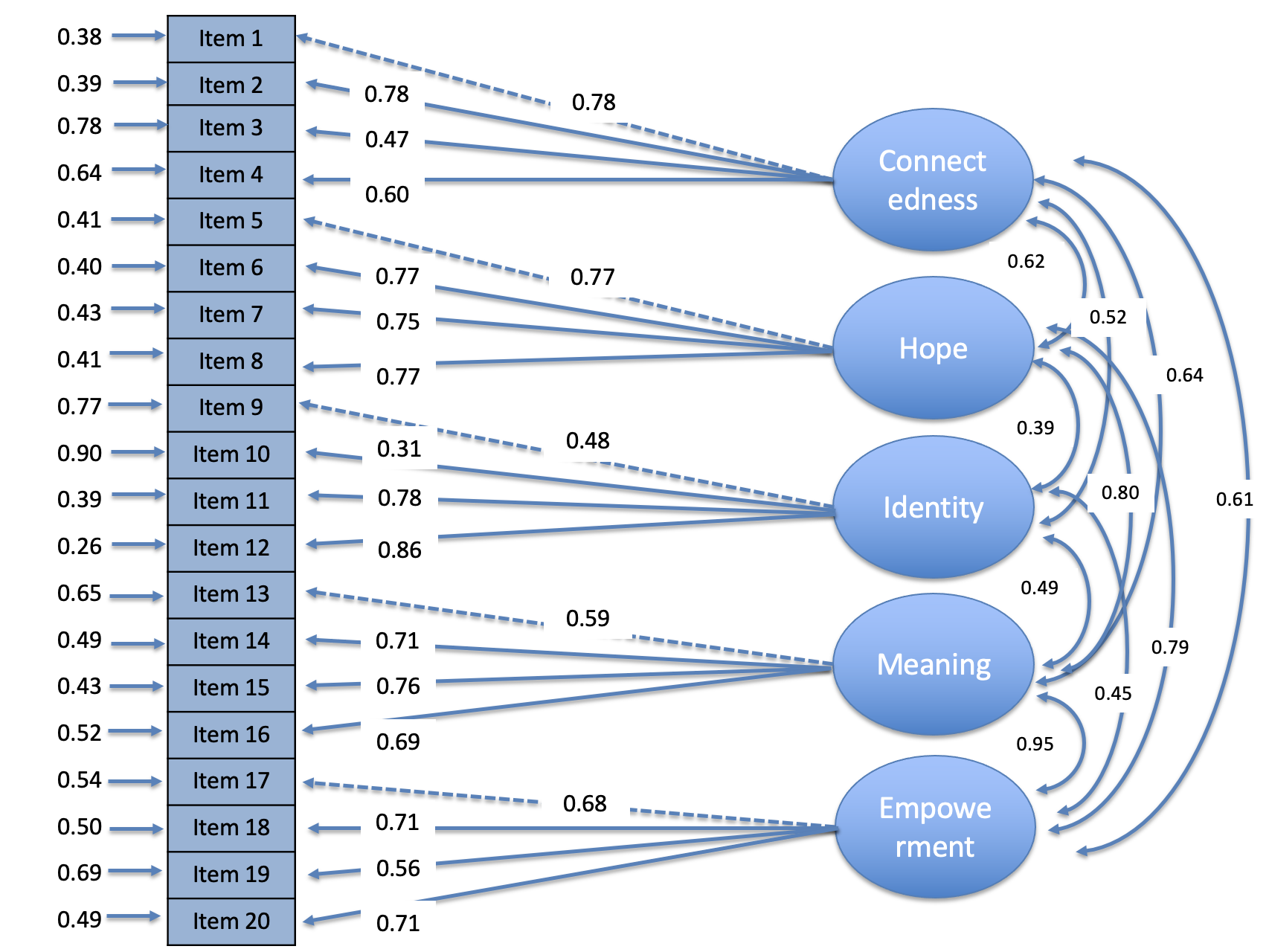** |
| --- |
| **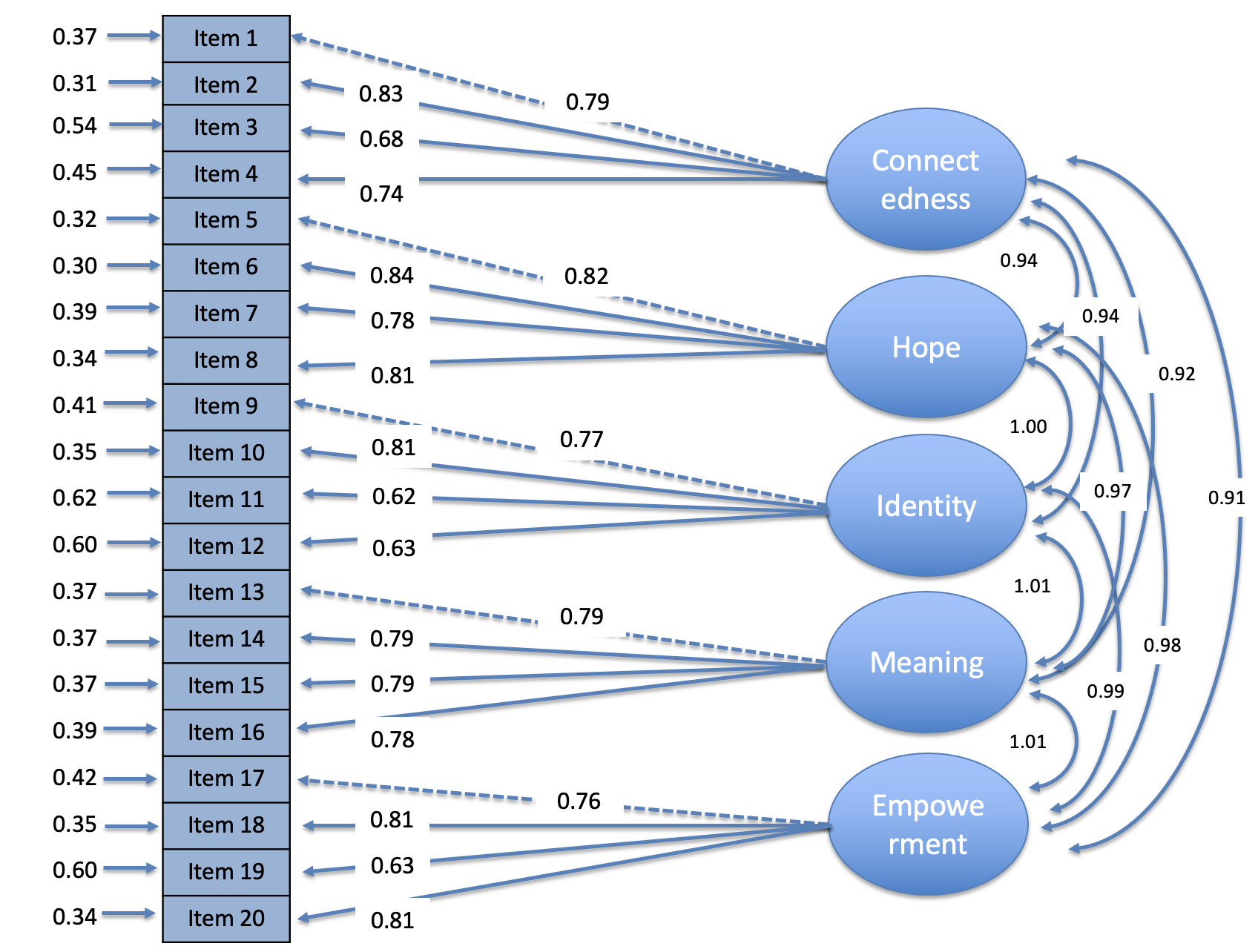** |
